# Understanding complex functional wiring patterns in major depressive disorder through brain functional connectome

**DOI:** 10.1038/s41398-021-01646-7

**Published:** 2021-10-13

**Authors:** Zhiyun Yang, Lingyu Jian, Hui Qiu, Chaoqing Zhang, Song Cheng, Junjun Ji, Ting Li, Yu Wang, Junfeng Li, Kefeng Li

**Affiliations:** 1 Department of Radiology, Heping Hospital Affiliated to Changzhi Medical College, Changzhi, Shanxi China; 2 Graduate College, Jinan University, Guangzhou, Guangdong China; 3 Huaxi MR Research Center (HMRRC), Department of Radiology, West China Hospital of Sichuan University, Chengdu, Sichuan China; 4 Department of Psychiatry, Changzhi Mental Health Center, Changzhi, Shanxi China; 5 School of Medicine, University of California, San Diego, CA USA

**Keywords:** Diagnostic markers, Depression

## Abstract

Brain function relies on efficient communications between distinct brain systems. The pathology of major depressive disorder (MDD) damages functional brain networks, resulting in cognitive impairment. Here, we reviewed the associations between brain functional connectome changes and MDD pathogenesis. We also highlighted the utility of brain functional connectome for differentiating MDD from other similar psychiatric disorders, predicting recurrence and suicide attempts in MDD, and evaluating treatment responses. Converging evidence has now linked aberrant brain functional network organization in MDD to the dysregulation of neurotransmitter signaling and neuroplasticity, providing insights into the neurobiological mechanisms of the disease and antidepressant efficacy. Widespread connectome dysfunctions in MDD patients include multiple, large-scale brain networks as well as local disturbances in brain circuits associated with negative and positive valence systems and cognitive functions. Although the clinical utility of the brain functional connectome remains to be realized, recent findings provide further promise that research in this area may lead to improved diagnosis, treatments, and clinical outcomes of MDD.

## Introduction

Major depressive disorder (MDD) is a common psychiatric disorder characterized by significant persistent sadness and emotional changes [1]. The World Health Organization (WHO) estimated that nearly 300 million people worldwide suffered from depression as of 2015 [2]. The number of patients with depression is expected to be considerably increased in the post-COVID-19 era [3, 4]. In addition, depression is one of the leading causes of disability and suicide, with over 800,000 suicides each year globally [5]. These high morbidity and mortality figures highlight the need for the precise diagnosis and effective treatment of MDD.

In contrast to other fields of medicine, current diagnosis in MDD primarily relies on subjective symptoms and observable signs in combination with the Hamilton Depression Rating Scale (HAM-D) scores and Diagnostic and Statistical Manual of Mental Disorders (DSM-V) criteria [6]. Differential diagnoses of MDD and other mental disorders such as schizophrenia and bipolar disorder (BD), are challenging due to similar clinical symptoms and behaviors. It was reported that nearly 21% of patients with BD were misdiagnosed with MDD, which may cause significant potential consequences due to the ineffectiveness of standard antidepressant therapy for BD [7]. The diagnostic heterogeneity in MDD has motivated a renewed interest in developing new approaches for diagnosing depression.

The standard treatment for MDD includes antidepressants, electroconvulsive therapy (ECT), repeated transcranial magnetic stimulation (rTMS), acupuncture, and cognitive behavioral therapy (CBT). Responses to any given therapy for MDD are often patient-specific. For 30–40% of patients, no effective treatment is found after more than one year of testing different antidepressants using standard care and a trial-and-error process [8]. Moreover, more than one-third of remitted patients had recurrence within one year of discontinuation of treatment [9]. Common clinical variables as biomarkers have had poor performance for predicting MDD recurrence and treatment responses [10].

Magnetic resonance imaging (MRI) is becoming an important non-invasive tool to aid in the diagnosis and treatment of MDD due to its high resolution for soft tissue [11]. In particular, functional MRI (fMRI) detects brain neuronal activities by measuring fluctuations in blood oxygen levels, providing an opportunity to refine pathobiological models of MDD. The human brain is intrinsically organized into distinct, functionally coherent networks, and MDD is increasingly recognized as a disease of network dysfunction [12]. Identification of neural elements and the interconnections by fMRI, also called the connectome, is, therefore, an important step toward a complex system approach to understand aberrant communications between brain-wide networks in MDD [13]. The fMRI-based connectome may provide a clearer understanding of MDD-related pathophysiology than investigating altered brain structure or function alone [14, 15]. In the past decade, extensive efforts have been made to understand the brain connectome related to depression, with the human connectome project (HCP) being the most notable project [16].

In this review, we summarized our current knowledge (2000–2021) of the brain functional connectome for MDD. We manually searched electronic databases PubMed and Embase for English language articles published from January 1, 2000, to February 20, 2021. We used the following search terms in combination with the terms “depression”, “MRI” and “functional connectivity”: “bipolar disorder”, “schizophrenia”, “recurrence”, “suicide”, “antidepressant”, “cognitive behavioral therapy”, “electroconvulsive therapy”, “repetitive transcranial magnetic stimulation”, and “acupuncture”. The articles were selected based on the quality of the study, and large randomized controlled trials (RCTs), high-quality observational studies, and reviews were preferred. The case reports and articles in non-peer-reviewed journals were excluded.

We first described the workflow of the fMRI-based brain functional connectome and then compared the distinct patterns of the brain functional connectome between MDD and other similar clinical conditions. The later sections focused on the application of the brain functional connectome in predicting MDD recurrence and suicide risk. We also discussed the roles of the brain functional connectome in evaluating the mechanisms of various treatment approaches for MDD, including antidepressants, CBT, ECT, rTMS, and acupuncture. Finally, the conclusion and perspectives on fMRI-based brain functional connectome were presented.

## The workflow for assessing the fMRI-based brain functional connectome

**Fig. 1:**
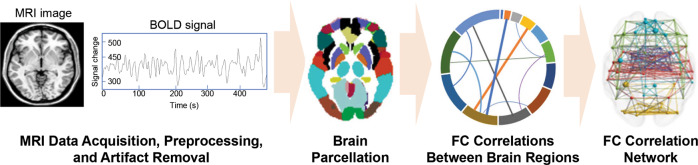
The brief workflow of fMRI-based brain functional connectome. FC functional connectivity, BOLD blood oxygen level dependent.

fMRI can map the brain functional connectome to characterize the information transmission between brain regions [16]. The blood oxygen level-dependent (BOLD) signals that underly most fMRI connectome applications are based on the agreement that the changes in blood flow and oxygenation are associated with neural activities. Figure 1 demonstrates the brief workflow of brain functional connectome including, including data acquisition at the high spatial and temporal resolution, preprocessing, brain parcellation, and correlation analysis [16]. We also listed the common software tools used in brain functional connectome analysis in Table 1.

**Table 1:** Software and tools commonly used in resting-state functional connectome analysis.

Software tools	URL	Main application
DPARSF	http://rfmri.org/DPARSF	Data preprocessing and statistical analysis
DPABI	http://rfmri.org/dpabi	Data preprocessing and statistical analysis
SPM12	http://www.fil.ion.ucl.ac.uk/spm	Data preprocessing and statistical analysis
restplus	http://resting-fmri.sourceforge.net	Data preprocessing and statistical analysis
Group ICA Toolbox	http://icatb.sourceforge.net/groupica.htm	Independent component analysis
GraphVar	http://rfmri.org/GraphVar	Graph theory analysis
GRETNA	https://www.nitrc.org/projects/gretna	Data preprocessing and graph theory analysis
Graph Theory GLM	https://www.nitrc.org/projects/metalab_gtg	Data preprocessing and graph theory analysis
CONN	https://www.nitrc.org/projects/conn	Data preprocessing and statistical analysis
C-PAC	https://fcp-indi.github.io/	Data preprocessing and statistical analysis
CosmoMVPA	http://www.cosmomvpa.org/	Multi-voxel pattern analysis
BrainNetViewer	https://www.nitrc.org/projects/bnv/	Visualization of results
MRIcro	https://www.nitrc.org/projects/mricro/	Visualization of results
MRIcron	https://www.nitrc.org/projects/mricron	Visualization of results
Xjview	https://www.alivelearn.net/xjview/	Visualization of results

First, whole-brain resting state fMRI is performed, and T1-weighted and T2-weighted images are obtained using the corresponding MRI sequences. fMRI data were also obtained by collecting BOLD signals of the whole brain. fMRI data are then preprocessed to minimize distortion, blurring, and temporal artifacts. To standardize the MRI data collection and preprocessing, in 2016, HCP published a detailed protocol for imaging data preprocessing (HCP-style paradigm) [16]. Second, brain parcellation is then conducted to define network nodes based on cytoarchitecture or anatomy. Due to recent advances in neuroimaging methodology, high-quality parcellations maps have been constructed, including more than 600 cortical and subcortical areas [17]. Either partial correlations or Pearson’s correlations are then computed between brain regions, which estimate direct connection strengths. The resultant correlation values are converted into z statistics with Fisher’s r-to-z transformation, resulting in a normally distributed connectivity matrix. The obtained brain functional connectome networks can then be analyzed by routine statistical analysis between regions of interest (ROIs). In recent years, researchers have also been exploring more robust, data-driven machine learning and graph theory algorithms such as independent component analysis (ICA), dynamic causal modeling (DCM), support vector machine (SVM), deep learning (DL), and graph theory analysis for feature selection and reduction, clinical outcomes prediction, and analysis of the importance of subnetworks in the brain, considering the whole connectivity among all brain areas [18–20]. Moreover, machine learning approaches, particularly artificial neural networks (Autobarcoder) and connectome-based predictive modeling (CPM), were demonstrated to play important roles in brain fingerprinting and provide insights in identifying individual-level functional abnormalities from whole-brain connectivity matrices [21, 22].

## Molecular mechanisms of MDD and brain functional connectome

**Fig. 2:**
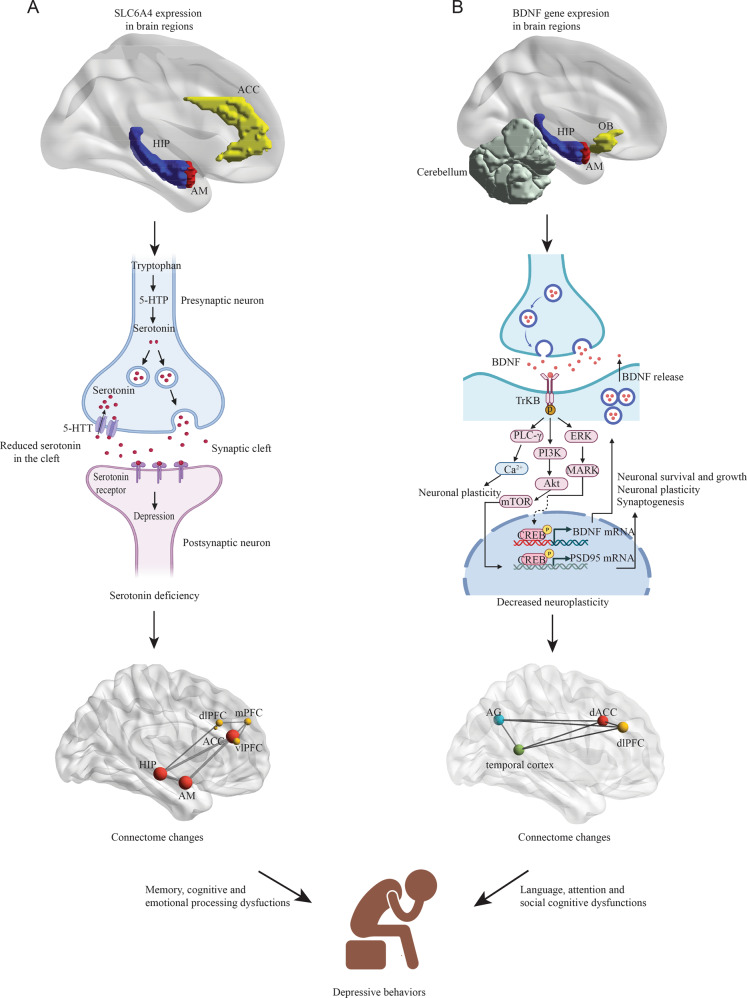
The associations between gene expression, MDD pathogenesis, and fMRI-based brain functional connectome changes. (**A**): Serotonin deficiency hypothesis; (**B**): Neuroplasticity hypothesis. The gene expression mappings were created according to PET-MR data from Ref. [26], and RNA-seq data from Ref. [25]. The colored brain regions represent higher gene expression levels. The maps of brain functional connectome were manually created according to the data from Ref. [33]. HIP, Hippocampus; AM, Amygdala; ACC, Anterior cingulate cortex; OB: Olfactory bulb; BDNF, Brain-derived neurotrophic factor; 5-HTTP, 5-Hydroxytryptophan; 5-HTT, Serotonin transporter; mPFC, medial prefrontal cortex; AG, Angular gyrus; dACC, Dorsal anterior cingulate cortex; dlPFC, dorsolateral prefrontal cortex.

Although several theories have been proposed for MDD pathogenesis, the serotonin hypothesis is still the most prevailing molecular mechanism of depression. This theory postulates that serotonin deficiency in the synaptic cleft is involved in the etiology of MDD, which is supported by the clinical efficacy of many current antidepressants such as serotonin selective reuptake inhibitors (SSRIs) [23]. Serotonin (5-hydroxytryptamine, 5-HT) in the brain is biosynthesized through tryptophan metabolism and stored within the vesicles in the presynaptic neurons at rest (Fig. 2A). Upon stimulation, serotonin is released to the synaptic cleft and binds to serotonin receptors on the postsynaptic neurons to produce normal brain functions. Previous studies highlighted the critical roles of 5-HT transporters (5-HTT) in serotonin transmission, and 5-HTT deletion in genetic mice models led to depressive-like behaviors [24].

5-HTT is a protein encoded by the SLC6A4 gene in humans. Although neuronal serotonergic cells project to virtually every brain area, 5-HTT availability is not equally distributed throughout the brain. Recent studies based on RNA sequencing and positron emission tomography (PET) imaging showed that the SLC6A4 gene is highly expressed in subcortical limbic regions of the human brain, specifically the hippocampus, amygdala, and anterior cingulate cortex (ACC), which are the network hubs of the human functional connectome related to emotional processing, cognitive ability, and memory [25, 26]. As the “bottle-neck” for the termination of neural serotonin actions, 5-HTT dysregulations could affect brain network connectivity associated with these susceptible brain areas and subsequently cause depressive symptoms in MDD patients (Fig. 2A). A number of studies had investigated the influence of genetic polymorphisms of 5-HTT on brain functional connectome in the context of MDD [27–30]. The consensus obtained from previous studies is that the brain functional connectome demonstrates tight coupling with the transcriptional activity of the SLC6A4 gene. In addition, the results supported the hypothesis that network connectivity failure in MDD starts in the hippocampus, amygdala, and ACC and spread to other brain areas through network hubs.

Besides the monoamine hypothesis, it has been suggested that impaired neuroplasticity may be causally associated with the development and course of depression. The neuroplasticity hypothesis of depression is supported by the evidence of decreased concentration of brain-derived neurotrophic factor (BDNF) in both animal models of depression and patients with depression. BDNF in the brain is a common neurotrophin that play important roles in synaptic plasticity and neuronal survival through binding TrkB receptors in postsynaptic neurons [31]. Upon binding to BDNF, TrkB becomes phosphorylated, which leads to activation of several downstream signaling pathways such as PLC-γ/IP3, PI3K/Akt, and MARK/ERK (Fig. 2B).

Among different brain regions, BDNF exhibits its highest expression in the hippocampus, amygdala, olfactory bulb, and cerebellum. The hemizygosity of BDNF modifies the availability of BDNF in the synaptic cleft. Similar to serotonin signaling, the single nucleotide polymorphism (rs6265) of BDNF, also known as Val66Met, had been demonstrated to reduce not only the hippocampal volume but also brain functional connectome crucial for language, attention, and emotions [32, 33]. For example, Val66Met BDNF was correlated with reduced functional connectivity involving the bilateral hippocampus, temporal cortex, dorsal anterior cingulate cortex (dACC), and dorsolateral prefrontal cortex (dlPFC) [34, 35].

## Brain functional connectome for differentiating MDD from other diseases

### MDD versus bipolar disorder (BD)

The misdiagnosis rate of BD patients is extremely high due to the similarities in the clinical presentation of depressive episodes present in both unipolar and bipolar depression and the higher prevalence of unipolar depression compared with BD [36], Misdiagnosing BD can cause deleterious consequences of inappropriate medication treatment, and it is particularly important to find objective approaches to distinguish unipolar patients from BD with high accuracy [37, 38]. The brain connectome has been used to explore differences in FC between BD and MDD patients, and the two groups have been robustly differentiated by alterations in large-scale functional networks, also known as intrinsic brain networks [39, 40] (Box 1).

**Fig. 3:**
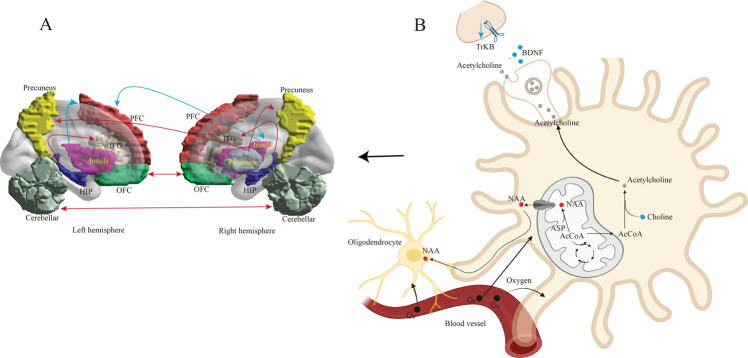
The relationship between neurometabolic changes and alternations of brain functional connectome in bipolar disorder (BD) compared to MDD. **A** Differences in brain functional connectome between BD and MDD. The red connections indicated increased functional connectivity (FC) between two brain regions in patients with BD, and the blue connections indicated decreased FC in BD patients. **B** Changes of brain neurotransmitters and their receptors and BDNF in ACC, right prefrontal lobe, and OFC of BD patients compared to MDD. Blue dots and arrows indicated decreased levels, and red dots indicated increased levels in BD patients compared to MDD. IFG, inferior frontal gyrus; OFC, orbitofrontal cortex; PFC, prefrontal cortex; HIP, hippocampus; NAA, N-Acetylaspartic acid; BDNF, brain-derived neurotrophic factor.

The frontoparietal network (FPN), also known as the central executive network (CEN), is involved in working memory and sustained attention. Compared to unipolar patients, BD patients showed significantly increased FC within the FPN, primarily in the left ventrolateral and dorsolateral prefrontal cortex (vlPFC and dlPFC) [39]. The hippocampus plays important roles in emotion regulation, learning, and consolidating memory and has been implicated in the pathophysiology of both MDD and BD [41]. In comparison with MDD patients, BD patients displayed increased FC between the bilateral posterior hippocampi and inferior frontal gyri (IFG) (Fig. 3A) [42]. FC differences in the hippocampus may cause differential emotional regulation between BD and MDD. In addition, FC between the right putamen and bilateral precuneus was significantly increased in patients with BD compared with MDD, resulting in an area under the receiver operating characteristic curve (AUROC) of 0.81 for the differentiation between these two kinds of patients (Fig. 3A) [43]. The precuneus is a core area of the default mode network (DMN), while the putamen belongs to the salience network (SN). FC between the right putamen and bilateral precuneus was correlated with emotion regulation. The abilities of emotion regulation in BD patients were less compromised than those in MDD patients regarding emotional awareness, acceptance of emotions, and understanding of emotions [44]. BD patients were also reported to display higher local FC than MDD patients within the bilateral orbitofrontal cortices (OFC) (Fig. 3A) [45]. At present, there are few reports about cerebellar FC changes. Wang et al., found that bilateral cerebellar FC was increased in patients with BD and decreased in patients with MDD compared to healthy controls [46].

In contrast, compared to unipolar MDD patients, BD patients had decreased FC in the DMN, primarily in the bilateral precuneus (PCUN) and hippocampi [39]. High FC in the DMN in MDD patients was associated with negative and ruminative thoughts [47]. Decreased FC was also reported in BD patients between the left insula and dlPFC and between the bilateral insula and the right frontopolar prefrontal cortex (FPPFC) (Fig. 3A) [48]. Further studies showed that FC in the somatosensory and motor cortices was decreased in all insular subregions in the BD group compared with the MDD group [49]. Overall, these studies indicated that studying the fMRI-based brain functional connectome might help in developing new imaging-based biomarkers that facilitate distinguishing BD from MDD at the group level.

In term of the biomarkers in other domains for differential diagnosis of BD and MDD, much of the interest was directed at neurotransmitters and their associated receptors as well as neurotrophic factors in brain regions such as ACC, right prefrontal lobe, and orbital frontal cortex (OFC). The most consistent results across several studies were the increased N-Acetylaspartic acid (NAA) and glutamate (Glu) levels in ACC and right prefrontal lobe in bipolar depression in contrast to unipolar [50, 51] (Fig. 3B). The choline level in ACC was reported to be lower in BD than that in MDD [51]. NAA and choline are well-known indicators of neuronal viability. Changes of NAA and choline in BD patients will alter the viability and oxygen utilization in neurons and oligodendrocytes and therefore induce FC alternations between the associated brain regions revealed by BOLD fMRI. Additionally, a few recent studies explored the BDNF level and its receptor TrKB expression in BD, which are related to synaptic plasticity [52, 53]. They found that BDNF and the expression of TrKB were significantly reduced in BD compared to unipolar depression (Fig. 3B). These findings support the theory that the differential functional network abnormalities and cognitive dysfunction between MDD, and BD are related to the disturbances of neurotransmitters and BNDF signaling.

Box 1 Three large-scale brain functional networks

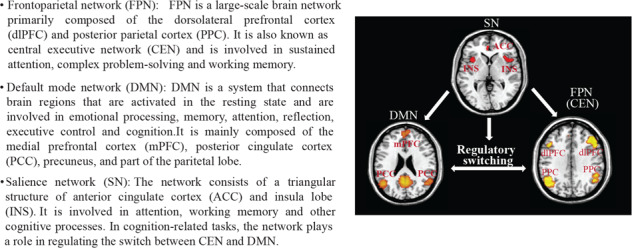

### MDD versus schizophrenia (SZ)

MDD is one of the most commonly misdiagnosed psychiatric disorders, and there is a high degree of overlap between the symptoms of MDD and SZ. A recent study reported that nearly 55% of patients with MDD in Ethiopia were more likely to be misdiagnosed as having SZ [54]. Differences in FC in large-scale brain networks and across various brain regions observed between MDD and SZ highlight the different pathophysiological mechanisms between these two disorders, which might be used for differential diagnosis.

In terms of large-scale networks, previous studies have identified distinct dysconnectivity patterns within the triple network framework, that includes the DMN, SN, and FPN. MDD patients were found to have stronger FC between the SN and DMN than SZ patients [55]. The most discriminative FC regarding the SN-DMN was between the fronto-insular SN and the posterior-parietal DMN [55]. Additionally, Jiang and the coauthors showed that the FC between the FPN-DMN was significantly increased in SZ patients and decreased in MDD patients compared to healthy controls (HCs) [56]. In contrast, MDD patients showed an increase in FC between the FPN-SN, while SZ patients displayed decreased FC compared to HCs [56]. Even though both SZ and MDD patients had negative FC between the left FPN, and the auditory network compared to HCs, MDD patients showed weaker FC than SZ patients between these two regions, which play roles in interaction with the environment [57].

Regarding various brain regions, FC differences were also observed in the frontal lobe and temporal lobe between SZ and MDD patients. The frontal lobe is involved in controlling important cognitive skills in humans, such as emotional expression and memory. FC within the prefrontal cortex (PFC) of the frontal lobe was significantly reduced in schizophrenia patients compared with MDD patients [58]. Wei and the coauthors analyzed FC between the PFC and the amygdala and found a decrease in FC in MDD patients, but not in SZ patients [59]. The temporoparietal junction (TPJ), a junction of the temporal and parietal lobes, plays roles in numerous aspects of social cognition. A recent study reported that FC between the right posterior TPJ and the left fusiform gyrus (FFG) as well as the right superior-posterior temporal cortex was decreased in patients with SZ compared with MDD patients [60]. Additionally, resting network connectivity analysis of thalamic nuclei identified reduced FC between the right pulvinar and right posterior cingulate cortex (PCC) and between the right mediodorsal nuclei (MDN) and right dorsal anterior cingulate cortex (dACC) in SZ patients compared to MDD patients [61].

## Brain functional connectome signatures for MDD recurrence

A high proportion of patients with remitted MDD will experience recurring episodes, and clinical symptoms tend to be more severe in recurrent episodes than in previous episodes [62, 63]. Recurrent MDD patients were reported to have abnormal functional connectivity compared to single-episode MDD patients and healthy controls. A recent study found higher connectivity in the right superior anterior temporal lobe (SATL) to the subgenual cingulate cortex, adjacent septal region, right ventral putamen, and temporoparietal junction (TPJ) in the recurrent MDD group than in the single-episode MDD and the HC groups [64]. However, the default mode network in patients with recurrent MDD had fewer connections to other subnetworks than HCs [65]. Additionally, decreased interhemispheric left-to-right subgenual anterior cingulate cortex (sgACC) connectivity distinguished MDD patients with recurring episodes from remitted MDD controls [66].

## Brain functional connectome for predicting suicide risks in MDD patients

Suicide ideation and behavior are closely related to psychopathology, especially depression [67]. It is difficult to predict whether MDD patients will have suicidal ideation and attempts based on their clinical symptoms [68]. The fMRI-based connectome provides a useful approach to explore the neurocircuitry basis of suicidal ideation in MDD patients.

**Fig. 4:**
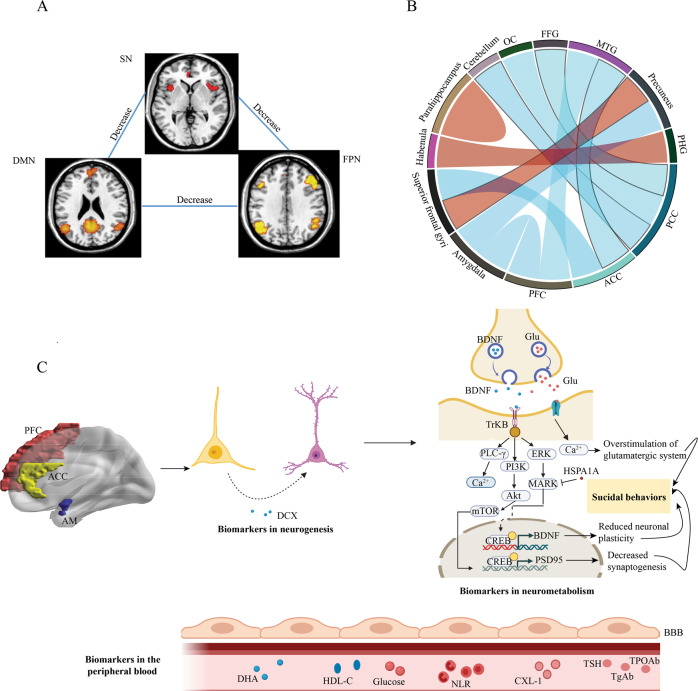
Predictive biomarkers to differentiate MDD patients with and without suicidal attempts. **A** Functional connectivity (FC) differences in large-scale brain networks. The green line indicated the significant decrease of FC in MDD with suicidal ideation compared to MDD without suicidal attempts. **B** Increase (red color connections) or decrease (blue color connections) of FC changes in local brain regions for MDD patients with suicidal attempts compared to MDD without suicidal attempts. **C** Protein and metabolite biomarkers in brain regions and peripheral blood for the differentiation of MDD patients with and without suicidal ideation. Red dots indicated increased expression or concentration in MDD with suicidal ideation compared to MDD without suicidal attempts. Blue dots indicated decreased expression or concentration. SN, Salience network; DMN, Default mode network; FPN, Frontoparietal network; OC, occipital cortex; FFG, fusiform gyrus; MTG, middle temporal gyrus; PHG, parahippocampal gyrus; PCC, posterior cingulate cortex; ACC, anterior cingulate cortex; PFC, Prefrontal cortex; AM, amygdala; DCX, Doublecortin; BDNF, Brain-derived neurotrophic factor, Glu, Glutamate; HSPA1A, Heat shock protein family A member A; DHA, Docosahexaenoic acid; HDL-C, High-density lipoprotein cholesterol; NLR, Neutrophil-to-lymphocyte ratio; TSH, thyroid-stimulating hormone; TgAb, anti-thyroglobulin; TPOAb, thyroid peroxidases antibody; BBB, blood–brain barrier.

Hypoconnectivity has been observed between large-scale brain networks, especially those involved in executive functions, in both adult and adolescent MDD patients with suicidal ideation compared with those without suicidal attempts (Fig. 4A). For instance, a recent study reported that decreased FC between the left FPN, anterior DMN, and SN was associated with greater severity of lifetime suicidal ideation [69]. Similarly, hypoconnectivity was observed between the anterior DMN and right FPN in young depressed patients with suicide attempts [70]. The PCC forms a central node in the DMN and has been implicated as a key part of human awareness. Interestingly, suicidal ideation scores were found to be positively correlated with decreased FC between the left PCC-left cerebellum, left PCC-lateral occipital lobe, and left PCC-FFG in adolescents with depression [71].

Differential functional connectivity in brain circuits related to cognitive functions has also been observed between MDD patients with and without suicide attempts (Fig. 4B). Fronto-limbic connectivity was demonstrated to be involved in the regulation of emotion [72]. The anterior cingulate cortex (ACC) is the frontal part of the cingulate cortex, with connections to both the “emotional” limbic system and the “cognitive” PFC [73]. Du and coauthors found decreased FC between the rostral anterior cingulate cortex (rACC), orbital medial PFC (OMPFC) and right middle temporal gyrus in MDD patients with suicidal ideation compared with healthy controls and MDD patients without suicidal ideation [74]. FC between the pregenual ACC (pgACC) and superior frontal gyri was also significantly decreased in adolescents with MDD and suicidal ideation and was positively correlated with suicidal ideation scores [75]. Women have been found to have a disproportionately higher rate of suicide attempts than men [76]. A recent study analyzed the FC of the amygdala, a key brain region involved in emotional and cognitive processing in female patients with MDD and suicidal ideation [77]. The authors identified the unique FC abnormality between the amygdala and precuneus/cuneus in female MDD patients with suicidal ideation [77]. Additionally, there was diminished FC between the bilateral amygdala and ventromedial prefrontal cortex (vmPFC), in both male and female MDD patients with suicidal attempts compared with individuals who had MDD without a history of suicidal attempts and healthy controls [78].

Several studies also reported brain region-dependent hyperconnectivity in MDD patients with suicidal ideation. Resting-state FC between the superior frontal gyri and left precuneus was found to be positively correlated with suicidality [71]. In addition, MDD patients with suicide-related behaviors displayed increased resting-state FC between the left habenula and the left parahippocampal gyrus (PHG), which may mediate functional abnormalities in critical survival mechanisms, by which the habenula works as a suppressor of motor activity [79]. The activity of the parahippocampal gyrus has been reported to be associated with negative emotional experiences [80]. A recent resting-state fMRI study found that the connectivity between the anterior division of the right parahippocampus and the posterior division of the left parahippocampus was significantly increased in MDD patients with suicide attempts [81]. Graph theory analysis on fMRI data reveals valuable information about the topological architecture of brain connectome networks such as centralized hubs and modular organization without the seed selection process [82]. Using a graph-based voxel-wise FC mapping, Chen and the co-authors identified significantly higher FC strength in right OFC and the dorsomedial prefrontal cortex (dmPFC), two DMN nodes, in suicide attempters comparing to MDD patients without suicide attempts [83]. These findings highlighted the potential roles of OFC and dmPFC, impairments in suicidal behaviors.

Machine learning approaches can process high dimensional variables and assess all the brain compartments simultaneously [84]. A recent study developed a classification model using SVM and structural MRI data to identify suicide attempters in adolescent MDD patients with an accuracy of 78.6% and sensitivity of 73.2% [85]. Similarly, a deep learning method based on structural MRI was able to detect differential suicidality in depressive patients from depression to suicidal ideation and attempted suicide [86]. Dai and the co-authors found that the self-organizing data analysis technique (ISODATA), a semi-supervised machine learning classification model, could better predict the gradual susceptibility of suicide in MDD patients than the traditional statistical methods and the top classifiers were the functional connectivity within the frontal-temporal circuit [87]. Even though the application of machine learning to predict suicide attempts and suicidal ideation remain limited, the existing studies demonstrated that machine learning algorithms have the potential to identify individual suicide risks and thus improve suicide prevention among the high-risk patient population.

Interestingly, previous studies also reported some protein and metabolite biomarkers for suicidal behaviors in the brain regions with functional connectome alternations and the peripheral blood [88–92]. These proteins, neurotransmitters, and metabolites are primarily related to neurogenesis, neurometabolism, synaptic plasticity, and immune-inflammatory processes (Fig. 4C). The glutamate level in ACC was one of the neurometabolic biomarker that was highlighted because of its capacity of distinguishing MDD with and without suicidal sedation and being adequately reproduced by more than one study. The ACC is known to mediate the input from executive functions and motivational drives through its prefrontal and limbic projections [93]. Overstimulation of glutamatergic system in ACC led to suicide attempts in depressed adolescents [92].

## Brain functional connectome for evaluating treatment efficacy

### Antidepressant medications

For a long time, drug therapy has been the first choice of treatment for depression because of its advantages, such as simplicity and strong operability [94]. Brain connectivity fMRI analyses play essential roles in evaluating the efficacy of antidepressants, elucidating drug targets and pharmacological mechanisms of action, and predicting treatment responses (Table 2) [95].

**Table 2:** Changes of brain functional connectome in MDD patients in response to antidepressant treatment.

Antidepressants or classes	Functional connectivity (FC)	Changes of FC after treatment	Therapeutic or Predictive
SSRIs	Amygdala and right PFC	Decreased	Therapeutic
SSRIs	Amygdala and left PFC	Decreased	Therapeutic
SNRIs	Amygdala and OFC	Increased	Therapeutic
Ketamine	sgACC and hippocampus	Decreased	Therapeutic
Ketamine	Right PFC and left dACC	Decreased	Therapeutic
Sertraline	Within DMN	Increased	Predictive
Sertraline	DMN and FPN	Increased	Predictive
Sertraline	IFG and FEF	Increased	Predictive
Sertraline	Right AI and left PFC	Decreased	Predictive

*SSRIs* Selective serotonin reuptake inhibitors, *SNRIs* serotonin and norepinephrine reuptake inhibitors, *PFC* Prefrontal Cortex, *OFC* Orbitofrontal cortex, *sgACC* Subgenual anterior cingulate cortex, *dACC* Dorsal anterior cingulate cortex, *DMN* Default model network, *FPN* Frontoparietal network, *IFG* Inferior frontal gyrus, *FEF* Frontal eye field, *AI* Anterior insula.

The amygdala, located in the medial temporal lobe, is a critical brain region of emotion regulation. SSRIs including fluoxetine, sertraline, and citalopram, are among the most commonly prescribed antidepressants. It was reported that 8 weeks of SSRI treatment normalized the increase in functional connectivity between the amygdala and bilateral PFC in MDD patients [96]. Venlafaxine is another commonly prescribed antidepressant medication belonging to the serotonin and norepinephrine reuptake inhibitor (SNRI) class. Reduced resting‐state functional connectivity between the amygdala and OFC was observed in patients with MDD compared to healthy controls [97, 98]. After 8 weeks of venlafaxine treatment, dysregulated amygdala-OFC functional connectivity in the left cerebral hemisphere was corrected, which was significantly associated with clinical improvements in MDD patients [99].

Although SSRIs and SNRIs have dominated the treatment of depression, a major limitation of these antidepressants is the delayed clinical onset [100]. Ketamine has gained remarkable interest as an emerging treatment for treatment‐resistant depression (TRD) due to its rapid antidepressant properties, which can appear as early as several hours after administration [101]. A number of fMRI-based connectome studies have been conducted to elucidate the molecular mechanisms of ketamine’s effects on the brain. The frontostriatal circuits are essential in executive and psychomotor functions [102]. Patients with TRD showed increased FC within frontostriatal circuits in response to ketamine treatment [103, 104]. However, the ketamine-induced increase in FC within frontostriatal circuits was not replicated by a recent study [105]. Several factors may account for these inconsistent results, including longer posttreatment scan intervals, different scanners, or subject-related variables [105]. The human subgenual anterior cingulate cortex (sgACC) is an extensively connected component of the limbic system that is involved in emotion processing [73], anhedonic reward [106], and anticipation [107]. Hyperconnectivity between the sgACC and hippocampus has been observed in MDD patients due to excessive hippocampal glutamatergic afferents to the sgACC [108]. Ketamine treatment was reported to rapidly reduce sgACC hyperactivity to positive incentives [109]. Increasing evidence has suggested that infusions of a subanesthetic dose of ketamine also produce rapid antisuicidal effects in patients with TRD [110]. In a recent double-blind, placebo-controlled, randomized clinical trial, the authors revealed that PFC-related circuit modulation is essential for the antisuicidal effects of ketamine [111]. Ketamine infusion led to a reduction in hyperconnectivity between the right dlPFC) and the left dorsal anterior cingulate cortex (dACC) with more dramatic effects in the low-dose group (0.2 mg/kg) than in the standard-dose group (0.5 mg/kg) [111].

Treatment responses to SSRIs and SNRIs vary greatly between patients [112]. The fMRI-based connectome has a high potential to predict responses to therapy since functional connectivity and alterations in network characteristics might offer signals similar to biological brain functions [113]. Predictive biomarkers of a specific class of antidepressants would help stratify patients for tailored therapies and shorten the duration of depression. In a recent large fMRI trial with sertraline, higher FC within the DMN and between the DMN and FPN predicted better outcomes of sertraline [114]. In another study of old MDD patients (> 60 years old), a better response to sertraline was found to be associated with greater connectivity between the left pars triangularis of the IFG and the left frontal eye field (FEF) located in the frontal cortex [115]. Recently, Yuan and coauthors found that decreased resting-state FC between the right anterior insula and the left PFC was linked to early symptom improvement by sertraline treatment, including improvements in self-perceptual anxiety, somatic symptoms, and insomnia [116]. Interestingly, these treatment-related brain connectivity changes by SSRIs might be dependent on the serum TNF-α level in MDD patients [117].

### Cognitive behavioral therapy (CBT)

CBT is a type of time-limited, intensive, symptom-focused psychotherapy that aims to alleviate depressive symptoms by encouraging patients to change destructive or disturbing thought patterns that have negative influences on behavior and emotions [118]. Studies have shown that CBT has benefits similar to second-generation antidepressants, with lower rates of depression relapse and fewer side effects [119].

**Fig. 5:**
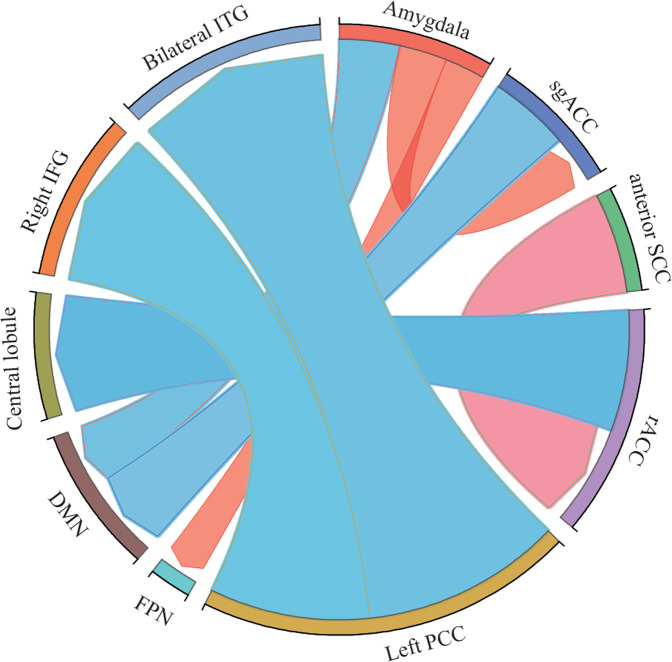
Changes of brain functional connectome in MDD patients in response to cognitive behavioral therapy (CBT) treatment. Blue color indicated decreased functional connectivity after CBT treatment, while red color indicated increased functional connectivity after CBT treatment. sgACC, Subgenual anterior cingulate cortex; SCC, Subcallosal cingulate; rACC, rostral anterior cingulate cortex; PCC, Posterior cingulate cortex; FPN, Frontoparietal network; DMN: Default mode network; IFG, inferior frontal gyrus; ITG, inferior temporal gyrus.

To date, CBT treatment has been primarily found to be associated with amygdala resting-state connectivity (Fig. 5). Studies have reported that CBT led to a significant increase in connectivity between the amygdala and brain regions that are involved in cognitive control, including the amygdala—FPN, and amygdala—sgACC [120, 121]. In contrast, amygdala and sgACC connectivity with regions of the DMN was reduced following CBT treatment [122].

The subcallosal cingulate (SCC) is known to be involved in top-down emotion regulation [123]. The effects of CBT interventions on the functional connectivity of the SCC remain largely unknown. Pantazatos et al. reported that after 14 courses of CBT treatment, resting-state functional connectivity between the anterior SCC and rACC dramatically increased in MDD patients., while rACC-central lobule connectivity decreased [124]. Their results highlighted the involvement of SCC subdivisions in cognitive interventions. Additionally, a recent study investigated the effects of rumination-focused CBT (RFCBT) on preventing the relapse of depression in adolescents with a history of MDD [125]. Interestingly, adolescents who received RFCBT demonstrated reduced rumination, which might have been associated with significant decreases in FC between the left PCC and the right inferior frontal gyrus (IFG) and bilateral inferior temporal gyri (ITG) [125].

### Electroconvulsive therapy (ECT)

**Fig. 6:**
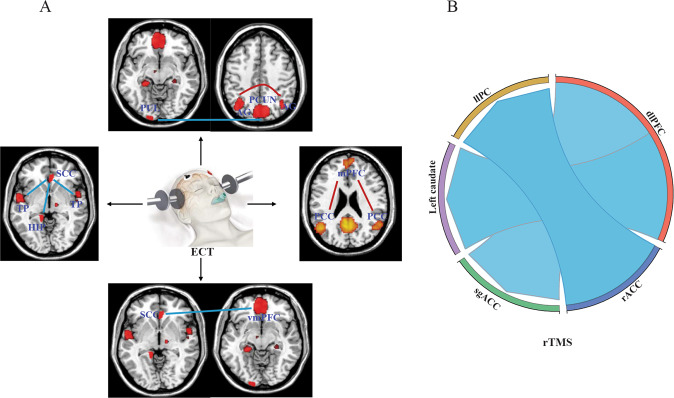
Changes of brain functional connectome in MDD patients in response to electroconvulsive therapy (ECT). **A** and repeated transcranial magnetic stimulation (rTMS). **B** Blue lines indicated decreased functional connectivity (FC) after treatment and red lines indicated increased FC after treatment. PUL, Pulvinar; PCUN, Precuneus; SCC, Subcallosal cingulate cortex; TP, Temporal polar; HIP, Hippocampus; vmPFC, Ventromedial prefrontal cortex; mPFC, Medial prefrontal cortex; PCC, Posterior cingulate cortex; AG, Angular gyrus; llPC, left lateral parietal cortex; dlPFC, dorsolateral prefrontal cortex; rACC, rostral anterior cingulate cortex; sgACC, Subgenual anterior cingulate cortex.

Electroconvulsive therapy (ECT) is a common approach for the treatment of MDD, especially for patients with refractory or severe depression [126], in whom the remission rate can reach as high as 50–80% by ECT treatment [127]. ECT delivers pulses of electricity to the brain through electrodes to treat depression. Previous studies have explored the neurobiological mechanisms underlying the therapeutic efficacy and the potential side effects of ECT in MDD patients using fMRI-based connectome analysis [128–130] (Fig. 6A).

By analyzing resting-state fMRI data from 84 subjects, Bai et al. found that ECT treatment restored the reduction in functional connectivity between dmPFC and PCC in MDD patients. Increases in FC between these two brain regions following ECT were correlated with improvements in depressive symptoms in MDD patients [131]. Additionally, one month of ECT treatment was reported to increase FC within the left angular gyrus (AG) of MDD patients, accompanied by improvements in mood and cognitive function [132]. FC between the SCC and the bilateral hippocampi, bilateral temporal polar cortices (TP), and vmPFC was significantly reduced in patients with TRD after ECT treatment [133].

Disruptions in functional interactions within the cognitive emotion regulation network (ERN) is one of the core features and underlying mechanisms of MDD [134]. A recent study evaluated the changes in functional connectivity between four submodules of the ERN, including the emotion response module (ERM), emotion integration module (EIM), emotion generation module (EGM), and emotion execution module (EEM) [135]. FC within the EEM, and intermodule FC between the EEM and EIM or ERM in MDD patients was dramatically increased following 15 days of ECT, which correlated with improvements in clinical symptoms in MDD patients [135]. These results indicated that the therapeutic efficacy of ECT is associated with the functional reorganization of intra- and intermodules within the ERN [135].

Although ECT is generally safe, it may cause memory loss, confusion and low verbal frequency [136]. These symptoms of cognitive impairment might be associated with decreased functional connectivity between the left pulvinar and bilateral precuneus following ECT [137].

### Repetitive transcranial magnetic stimulation (rTMS)

rTMS is a non-invasive treatment that uses specially designed magnetic coils placed near the head to electrically stimulate the brain to trigger neuronal action potentials by generating a rapidly changing strong magnetic field [138, 139]. rTMS has been approved by the U.S. Food and Drug Administration (FDA) as a therapeutic tool to treat severe depression because of its ability to specifically modulate distinct brain areas [140].

Clinical improvements for patients with TRD following rTMS were found to be related to the decreased functional connectivity between the sgACC and right dlPFC and increased connectivity between the rACC and left lateral parietal cortex (llPC) (Fig. 6B) [141]. It was also reported that 2 weeks of high frequency rTMS treatment led to a significant reduction in connection strength between the dlPFC and the left caudate nucleus (CN) in MDD patients, and these decreases predicted improvements in depressive symptoms [142].

### Acupuncture treatment

**Fig. 7:**
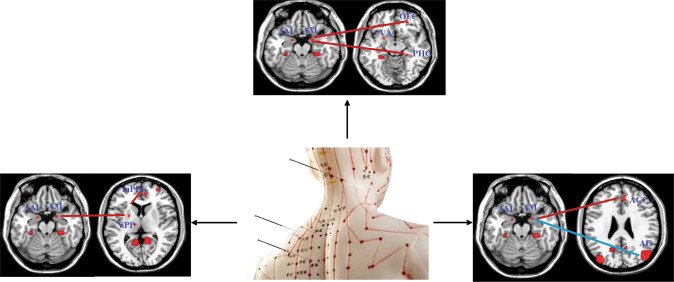
Changes of brain functional connectome in MDD patients in response to acupuncture treatment. Blue lines indicated decreased functional connectivity (FC) after treatment and red lines indicated increased FC after treatment. AM, Amygdala; mPFC, Medial prefrontal cortex; VPP, Ventral prefrontal putamen; ACC, Anterior cingulate cortex; AG, Angular gyrus; PHG, Parahippocampal gyrus; OFC, Orbitofrontal cortex.

The effectiveness and safety of acupuncture treatment, an essential therapeutic modality in complementary and alternative medicine, for depression have been confirmed in a number of studies [143–145]. fMRI-based brain connectome provides a useful tool to gather insights into acupuncture-related neural responses and the modulatory mechanisms underlying acupuncture (Fig. 7).

The amygdala and OFC are two key regions in the emotional regulatory nervous system. Dugan et al. found that acupuncture stimulation at BaiHui increased functional connectivity between the amygdala and the OFC and decreased FC between the amygdala and the angular gyrus in MDD patients [146]. Repeated acupuncture treatment also led to an increase in functional connectivity between the left amygdala and anterior cingulate cortex (ACC), and between the right amygdala and right parahippocampal gyrus (PHG) following acupuncture in combination with fluoxetine, which was significantly correlated with HAM-D scores [147].

A small clinical trial with 46 patients also showed that acupuncture treatment resulted in an increase in FC in brain regions involved in reward processing, such as FC between the ventral striatum and mPFC, and between the amygdala and ventral prefrontal putamen (VPP) [148]. At the end of 8 weeks of treatment, increased resting-state functional connectivity was significantly correlated with decreased depression severity scores [148].

## Conclusions and perspective

The development of fMRI-based brain functional connectome techniques has enabled us to navigate the living human brain in a way that was never before possible. Through analyses of the brain functional connectome, we can compare the brain circuits between MDD and other conditions and then zoom into the differential brain circuits to explore the functions that depend on them. Studies published in the past 20 years have demonstrated that connectome dysfunctions between brain circuits associated with negative/positive valence systems, cognitive systems, and cross-cutting elements are the core features of MDD (Table 3). The systematic exploration of large-scale functional brain networks plays promising roles in differentiating MDD from other psychiatric disorders, predicting MDD recurrence, suicide attempts, and treatment efficacy, and evaluating the mechanisms of treatment.

**Table 3:** The negative/positive valence systems and cognitive systems.

Systems	Behavior	Brain Circuits	Function
Negative valence systems	Threat (acute/potential); loss; frustration	Amygdala; hippocampus; insula; ACC; lateral PFC	Facial emotion identification
Positive valence systems	Reward (Responsiveness; learning; valuation)	Striatum; OFC; vmPFC; ACC	Positive emotion reactivity; reward responsiveness; reward learning
Cognitive systems	Working memory; cognitive control	DLPFC, DPC, precentral gyrus	Memory performance; concentration; impulsive behavior; speed of information processing; attention

*ACC* Anterior cingulate cortex, *PFC* Prefrontal cortex, *OFC* Orbitofrontal cortex, *vmPFC* Ventromedial prefrontal cortex, *DLPFC* Dorsolateral prefrontal cortex, *PCC* Posterior cingulate cortex, *AG* Angular gyrus, *amPFC* Anterior medial prefrontal cortex, *DPC* Dorsal parietal cortex.

Despite the substantial progress that has been made in the analysis of brain functional connectome, several technical issues remain unresolved. First, brain segmentation of function connectome is still challenging due to the complexity of the human brain and the absence of a true gold standard. Second, at present, most studies have not conducted in-depth optimization of the identification methods for time series characteristics of fMRI data. Third, there are discrepancies in using different algorithms to construct functional networks based on deep learning, and how to select algorithms to improve their applicability in the brain functional system remains to be studied. Furthermore, it is worth integrating multi-level templates and multi-modal images using machine learning approaches to enhance the accuracy and efficiency of large-scale brain connectivity.

Given the increasing public health need, we must improve our understanding of how connectome dysfunctions alter behaviors in MDD and the associations between connectome changes and the abnormalities of molecular biomarkers. Most past studies were limited by the small sample size. Connectome atlases based on a larger number of subjects per group and high-resolution imaging techniques will provide more reliable and consistent results. Connectome analysis could include more diverse age groups, especially adolescents. Additionally, the integration of results from different connectomes, including connectomes based on proteomics, metabolomics, transcriptomics and exposomics, may prove more informative than analyses based on a single connectome [149, 150].

In conclusion, alterations in the human brain functional connectome are central to the neurobiology of MDD and include changes in multiple, large-scale brain networks as well as local disturbances in brain circuits related to negative/positive valence systems and cognitive functions. With the growing compilation of brain connectome data, the development of high-resolution MRI imaging techniques, and the application of data-driven machine learning algorithms, we anticipate that brain connectome analyses will continue to surprise us and provide a better understanding of living healthy brains and MDD.
